# WNT-5a and SOST Levels in Gingival Crevicular Fluid Depend on the Inflammatory and Osteoclastogenic Activities of Periodontal Tissues

**DOI:** 10.3390/medicina57080788

**Published:** 2021-07-31

**Authors:** Georgios S. Chatzopoulos, Massimo Costalonga, Kim C. Mansky, Larry F. Wolff

**Affiliations:** 1 Division of Periodontology, Department of Developmental and Surgical Sciences, School of Dentistry, University of Minnesota, Minneapolis, MN 55455, USA; costa002@umn.edu (M.C.); wolff001@umn.edu (L.F.W.); 2 Division of Orthodontics, Department of Developmental and Surgical Sciences, School of Dentistry, University of Minnesota, Minneapolis, MN 55455, USA; kmansky@umn.edu

**Keywords:** gingival crevicular fluid, gingivitis, ELISA, periodontitis, sclerostin, Wnt

## Abstract

*Background and Objectives:* Wnt signaling leads to stimulation of osteoblasts and it reduces osteoclastogenesis and bone resorption via the regulation of the osteprotegrin and receptor activator of nuclear factor kappa-Β ligan (RANKL). Wnt signaling pathways are regulated by their physiological antagonists such as sclerostin (SOST) as well as WNT-5a. The aim of this study was to determine the total amount of Sclerostin and WNT-5a in the gingival crevicular fluid (GCF) in sites with a continuum from a healthy to diseased periodontium. *Materials and Methods:* In this cross-sectional study, a total of 20 patients with generalized periodontitis, 10 subjects with gingivitis as well as 14 individuals with a healthy periodontium were recruited upon clinical and radiographic periodontal examination. In patients diagnosed with periodontitis, GCF samples were collected from periodontitis, gingivitis and healthy sites, while gingivitis patients provided samples from gingivitis and healthy sites. In healthy patients, only healthy sites were sampled. Protein total amount of SOST and WNT-5a were quantified by sandwich enzyme-linked immunosorbent assay (ELISA). *Results:* A total of 108 GCF samples were collected from a total of 44 individuals. When all periodontitis (*n* = 51), gingivitis (*n* = 12) and healthy (*n* = 45) sites were analyzed regardless of the patient diagnosis, periodontitis sites demonstrated significantly elevated WNT-5a total amounts (*p* = 0.03) when compared to gingivitis sites. Gingivitis sites demonstrated a trend of more total SOST (*p* = 0.09) when compared to periodontitis and healthy sites. Within each patient diagnostic category, sites showed similar SOST and WNT-5a total amounts (*p* > 0.05). *Conclusions:* WNT-5a levels in GCF depend on the stage of periodontitis sites. SOST trended higher in the GCF of gingivitis sites but similar in chronic periodontitis and healthy sites. WNT-5a and SOST play a crucial role in periodontal tissue remodeling and depend on the inflammatory and osteoclastogenic activities.

## 1. Introduction

Periodontitis is defined as a chronic inflammatory disease that is induced by oral microorganisms in a susceptible host [1]. The colonization of the periodontal sulcus by symbionts, pathobionts and keystone bacteria, and the resulting immune response, leads to vascular proliferation, progressive destruction of alveolar bone, loss of connective tissue attachment and tooth loss, if left untreated [2]. Imbalance between the immune response and the microbiota is central to the pathogenesis of inflammation-induced bone loss [1]. The uncoupling of osteoclast-mediated bone resorption and osteoblast-mediated bone formation is a complex process of different cell types and control mechanisms [3]. Activation of a number of pathways plays a key role to promote or control the inflammatory response.

The Wnt pathway is a signal transduction pathway that regulates bone metabolism by controlling various cellular processes, including cell fate, cell polarity, cell migration and organogenesis [4]. The activation of the Wnt pathway is based on the interaction between Wnt proteins and transmembrane receptors that promote the expression of Wnt-dependent genes for the proliferation, differentiation, maturation and activity of osteoblast-lineage cells [5]. Wnts are a family of secreted glycoproteins that mediate signal transduction via canonical and noncanonical pathways. Pathways associated with Wnt signaling are regulated by their physiological antagonists such as Sclerostin (SOST) and WNT-5a that play important roles in the pathophysiology of several inflammatory conditions [6].

Sclerostin is an osteocyte-secreted glycoprotein encoded by the *Sost* gene that inhibits bone formation through the downregulation of the canonical Wnt/β-catenin cascade [7,8]. Sclerostin blocks Wnt proteins from binding to the extracellular regions of lipoprotein-receptor-related protein (LRP) 5/6 expressed on osteoblasts and, therefore, prevents osteoblast maturation into mineralizing bone cells [7,8]. In addition, Sclerostin promotes osteoclast differentiation and bone resorption by inducing the expression of the receptor activator of NF-kappa B ligand (RANKL) [9]. Strategies to neutralize Sclerostin with antibodies have been used for the treatment of osteoporosis and periodontal disease with promising results [10,11].

WNT-5a is a noncanonical Wnt-secreted glycoprotein that triggers proinflammatory signaling cascades. In comparison with SOST, which inhibits the canonical Wnt signaling pathway, WNT-5a is involved in the noncanonical Wnt pathway. This process increases the expression of proinflammatory cytokines and chemokines that are dependent on activation of the NF-κB pathway and inflammatory mediators in monocytes, macrophages, dendritic cells and microglia [12,13]. Thus, there is an interplay between Wnt signaling and inflammatory pathways, which supports the potential use of Wnt-5a as a novel prognostic or diagnostic biomarker for inflammatory diseases. Wnt-5a activates bone resorption by enhancing RANKL-induced osteoclastogenesis and promotes osteoblast differentiation through the upregulation of LRP5/6 [14,15]. WNT-5a can both activate (binding to frizzled receptor-4) and inhibit (activating the Ror-2 receptor) the canonical Wnt signaling pathway [16].

Diagnosis of periodontitis has traditionally involved clinical measurements of probing depth, attachment loss, evaluation of clinical signs of inflammation and oral hygiene performance and radiographic evaluation of the alveolar bone [17]. However, the use of clinical and radiographic tools can only lead to a retrospective diagnosis based on results of previous destruction while lacking a reliable and objective approach to diagnose and predict periodontal disease progression [17]. Diagnostics based on oral fluids, including saliva, oral rinses and gingival crevicular fluid (GCF), can be used in a noninvasive, independent and reliable way [18]. A more optimal diagnostic tool should provide information for the presence and severity of the disease and predict the future disease status and activity [19]. GCF is utilized to assess quantitatively the site-specific host-derived molecules in a reliable and less biased approach compared to other oral fluids [20]. A cross-sectional assessment of sites with different periodontal health statuses can identify a biomolecule as a diagnostic biomarker and is used extensively as an initial step to understand the roles of molecules in the periodontal inflammatory process. Both WNT-5a and SOST were expected to be present in GCF as they are expressed in periodontal tissues and in periodontal ligament [21,22].

To the best of our knowledge, there is no research comparing GCF levels of Sclerostin and WNT-5a in sites with chronic periodontitis, gingivitis and healthy periodontium. Considering the current knowledge in the field, we hypothesized that the total amount of Sclerostin and WNT-5a in GCF differ at sites with (i) chronic periodontitis, (ii) gingivitis and (iii) healthy periodontal tissues. The aim of this study was to determine the total amount of Sclerostin and WNT-5a in the GCF in sites with a continuum from a healthy to a diseased periodontium.

## 2. Materials and Methods

### 2.1. Study Design

This cross-sectional study consisted of 44 systemically healthy participants (28 females and 16 males; mean age of 42.5 ± 19.1 years); 20 with chronic periodontitis, 10 with gingivitis and 14 periodontally healthy individuals. Study participants were recruited from the pool of patients presenting to the Graduate Periodontology Clinic at the University of Minnesota School of Dentistry between February 2016 and September 2017. Individuals meeting the predefined inclusion and exclusion criteria were invited to participate in the study. The aims and the protocol of the research were explained, and written informed consent and Health Insurance Portability and Accountability Act (HIPAA) permission were obtained from all participants who agreed to enter the study. The study was approved by the Institutional Review Board (IRB) at the University of Minnesota (Study Number: 1511M80307; Approval Date: 22 August 2016).

### 2.2. Inclusion and Exclusion Criteria

Individuals were initially screened for eligibility for participation in the study based on specific inclusion and exclusion criteria that have been reported previously [23]. Briefly, participants should fulfill the following criteria (a) at least 18 years of age; (b) systemically healthy; (c) ≥15 permanent teeth (excluding third molars) present; and (d) absence of systemic conditions with a documented impact on periodontal status or bone-related diseases. Exclusion criteria were as follows: (a) periodontal treatment received in the last 12 months; (b) need for antibiotic premedication; (c) pregnancy or lactation; (d) antibiotic use in the last 6 months; (e) regular use of nonsteroidal anti-inflammatory drugs (NSAIDs); (f) diagnosis of aggressive periodontitis; (g) use of glucocorticoids, bisphosphonates or denosumab (anti-RANKL mAb); (h) periapical lesions; (i) orthodontic appliances; and (j) adults unable to provide consent.

### 2.3. Study Groups

Each participant underwent a full-mouth periodontal examination with radiographs to assess their periodontal status. Eligible individuals were categorized into periodontitis, gingivitis and healthy patient groups according to their periodontal status based on clinical (clinical attachment loss (CAL), probing pocket depth (PPD) and bleeding on probing (BOP)) and radiographic criteria (percentage of bone loss). In the periodontitis group, patients with generalized moderate and severe periodontitis (CAL ≥ 3 mm in over 30% of the sites, PPD ≥ 4 mm and bone loss of ≥40% in ≥30% of the sites) were included [24]. A full-mouth series of X-rays within the past 12 months was required to further assess the diagnosis of periodontitis. Adults with no clinical attachment loss or bone loss with BOP ≥ 10% were grouped as gingivitis patients, while individuals with BOP ≤ 10% were grouped as healthy patients [25]. A full-mouth series of intraoral radiographs within the last five years was needed to confirm the absence of radiographic periodontal bone destruction.

### 2.4. Clinical Examination

PPD (distance from the deepest point of the periodontal sulcus/pocket to the gingival margin), CAL (distance from the deepest point of the periodontal sulcus/pocket to the cementoenamel junction) and presence of plaque (modified Plaque Control Record by O’Leary et al. [26]) and BOP (modified Gingival Bleeding Index by Ainamo and Bay [27]) were recorded at six sites around each tooth using a Michigan “O” probe with Williams markings (Hu-Friedy, Chicago, IL, USA) by calibrated examiners (G.C., M.C.). All measurements were performed at six surfaces of each tooth: mesiobuccal, buccal, distobuccal, mesiolingual, lingual and distolingual. Both plaque and BOP were recorded as binary variables, either presence or absence. Percentage of sites with PPD = 1–3 mm, PPD = 4–6 mm and PPD ≥ 7 mm and percentage of sites with CAL = 0–2 mm, CAL = 3–4 mm and CAL ≥ 5 mm were also recorded. The calibrated examiner’s agreement for PPD was within ±1 mm, and for CAL, it was within ±2 mm, ranging from 92.1–100% to 92.9–100%, respectively. Calibration using a tolerance of a millimeter in pocket depth and two millimeters in attachment loss have been proposed in the literature [28,29].

### 2.5. Sample Collection

GCF sampling was performed either before full-mouth periodontal measurements or at least 2 weeks after the initial comprehensive examination by a single examiner (GC). After removing the supragingival plaque from the interproximal sites that were selected for sampling with a sterile hand instrument, a gentle stream of air was applied parallel to the buccal and lingual surfaces for 3–5 s with the use of an air syringe. Standardized sterile methylcellulose strips (Periopaper; ProFlow, Inc., Amityville, New York, NT, USA) were inserted, one at a time, into the gingival crevice until a mild resistance was felt. Each strip was held for 30 s in the sulcus, and if a strip was contaminated with blood or saliva, it was discarded. The GCF volume was measured with a precalibrated Periotron 6000 device (Oralflow Inc., Plainview, NY, USA). The strips from each participant were transferred in Eppendorf tubes and stored at −80 °C, until laboratory analysis. Each patient provided 1–3 samples. The number of samples per subject depended on the available sites fulfilling the predefined criteria of periodontitis, gingivitis, health.

In patients diagnosed with periodontitis, GCF samples were collected from: (i) periodontitis (PP) sites with PPD ≥ 5 mm, CAL ≥ 3 mm or PPD ≥ 4 mm with BOP (definition of unstable sites) [30]; (ii) gingivitis (PG) sites when PPD ≤ 3 mm with the presence of BOP (definition of sites in remission) [30]; and (iii) healthy (PH) sites when PPD < 4 mm without BOP (definition of stable sites). Gingivitis patients provided samples from: (i) gingivitis (GG) sites with no attachment/bone loss and presence of BOP and (ii) healthy (GH) sites when no attachment loss, no bone loss and absence of BOP were recorded. In healthy patients, only healthy (HH) sites with no attachment loss, no bone loss and absence of BOP were sampled. In addition, sites were grouped into periodontitis, gingivitis and healthy sites regardless of the patient group.

### 2.6. Laboratory Analysis

Sclerostin and WNT-5a were measured by an enzyme-linked immunosorbent assay (ELISA) using commercially available kits (Sclerostin/SOST: R&D Systems, Minneapolis, MN, USA; WNT-5a: Cusabio Biotech, Wuhan, China). Before the analysis, GCF samples were thawed from −80 °C for 30 min. Phosphate-buffered saline (PBS) (pH = 7.4) of 300 μL was added to each Eppendorf tube and left for 30 min. Then, the tubes were vortexed for 1 min and centrifuged at 906× *g* for 15 min at 4 °C. The supernatants were collected and assayed for total amounts of Sclerostin and WNT-5a following the instructions of the manufacturers. Quantities of Sclerostin, WNT-5a, were calculated based on the standard absorbance curve obtained from each assay plate and expressed as the “total amounts in 30 s” of collection time. The protein total amounts were measured in picograms for Sclerostin and in nanograms for WNT-5a.

### 2.7. Sample Size Calculation

The primary outcome variables (GCF levels of Sclerostin and WNT-5a) were used to calculate the appropriate sample size and determine the power of the study. This is the first study quantifying Sclerostin and WNT-5a GCF protein amounts in gingivitis sites, and therefore, the protein amounts were compared between periodontitis, gingivitis and healthy sites. Calculation of sample size was completed based on available data for periodontitis (generalized moderate and severe periodontitis) and healthy sites [23]. The sample size calculation was conducted based on the number of sites rather than patients due to the methodology of the present study. Assuming a pooled standard deviation of 0.9 ng for WNT-5a and 33.9 pg for Sclerostin, the study would require a sample size of 11 for each WNT-5a group and 5 for each Sclerostin group, to achieve a power of 80% and a level of significance of 5%, for detecting a true difference between the groups of 1.1 ng (WNT-5a) and 61.1 pg (Sclerostin).

### 2.8. Statistical Analysis

The normality of the distribution of the variables was examined by the Shapiro–Wilk test. The mean ± standard deviation (SD) was used to present the numerical variables. Counts and percentages were used for categorical variables. The age distribution of the subjects and the clinical measurements at the patient level were analyzed by one-way variance analysis (one-way ANOVA). To compare the categorical variables (gender, race and smoking habits), chi-square tests were performed. Generalized linear mixed models were used to compare the clinical and biochemical parameters between site groups and Bonferroni correction tests were utilized for post-hoc analysis. Comparisons between samples from the same patients was performed using the dependent *t*-test. Spearman’s rank correlation was used to test possible relationships between Sclerostin and WNT-5a gingival protein amounts and the clinical measurements at the sampled sites. All data were analyzed with SPSS v24 (SPSS Inc. IBM, USA). Values of *p* < 0.05 were considered statistically significant. Graphing was performed with GraphPad Prism software.

## 3. Results

### 3.1. Patient Demographics and Clinical Findings

The total population of the study consisted of 44 individuals (20 periodontitis, 10 gingivitis and 14 healthy) with a mean age of 42.5 ± 19.1 years. The mean age of the patients in the periodontitis group was significantly higher than those in the gingivitis and healthy groups (*p* < 0.001). Gender (*p* = 0.21) and race (*p* = 0.13) distributions were similar in the groups. Smoking habits were significantly different between the groups (*p* < 0.001), with current and ex-smokers being in the periodontitis group only. Both the gingivitis and healthy patient groups consisted of nonsmokers only (Table 1).

Missing teeth (*p* < 0.001), PPD (*p* < 0.001), CAL (*p* < 0.001), PI (*p* = 0.002), sites with PPD = 4–6 mm (*p* < 0.001), sites with CAL = 3–4 mm (*p* < 0.001) and sites with CAL ≥ 5 mm (*p* < 0.001) were significantly higher in the periodontitis patient group compared to the gingivitis and healthy groups. Sites with PPD = 1–3 mm (*p* < 0.001) were significantly lower in the periodontitis group than in the healthy and gingivitis groups. Sites with PPD ≥ 7 mm were significantly (*p* = 0.005) higher in the periodontitis group compared to gingivitis (*p* = 0.028) and healthy (*p* = 0.013). Full-mouth BOP frequency was significantly higher in periodontitis than in healthy subjects (*p* < 0.001) while BOP was similar in periodontitis and gingivitis subjects (*p* = 0.085).

### 3.2. Clinical Characteristics of Sampled Sites

A total of 51 periodontitis, 12 gingivitis and 45 healthy GCF samples were collected. Periodontitis sites were associated with significantly higher PPD, CAL, BOP, sites with PPD = 4–6 mm, PPD ≥ 7 mm, CAL = 3–4 mm and CAL ≥ 5 mm compared to gingivitis and healthy sites (*p* < 0.001). By definition, periodontitis sites did not include PPD = 1–3 mm and CAL = 0–2 mm (Table 2).

Forty-four individuals were sampled, collecting a total of 108 GCF samples. In patients diagnosed with periodontitis, we collected GCF from 51 periodontitis (PP), 6 gingivitis (PG) and 15 healthy (PH) selected sites. In patients diagnosed exclusively with gingivitis, we collected GCF from 6 gingivitis (GG) and 10 healthy (GH) sites, whereas in completely healthy patients, GCF was harvested from 20 sites (HH). The characteristics of those sites are reported in Table 3. PPD and CAL were significantly higher in the periodontitis sites compared to the gingivitis and healthy sites (*p* < 0.001). Sites with BOP were significantly higher in the periodontitis and gingivitis groups when compared to the healthy group (*p* < 0.001). Significant differences were also observed in regards to the distribution of the PPD and CAL clusters (*p* < 0.001). In the gingivitis patient group, gingivitis sites exhibited significantly higher BOP than the healthy sites (*p* < 0.001), while no other differences were found.

### 3.3. Total Protein Amounts of Periodontitis, Gingivitis and Healthy Sites

#### 3.3.1. Sclerostin Is Osteoclastogenic but Dampens Osteoblasts

**Figure 1:**
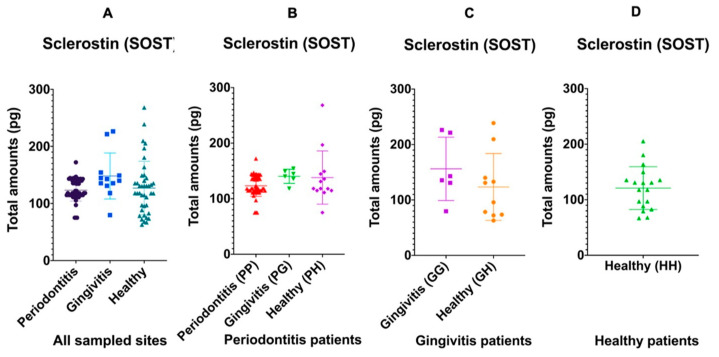
Distribution of the total amount of GCF Sclerostin in periodontitis, gingivitis and healthy sites regardless of the patient diagnosis (**A**); within the periodontitis patient group (**B**); gingivitis and healthy sites in the gingivitis patient group (**C**); and healthy sites in the healthy patient group (**D**). The solid lines show mean values.

When considering only the characteristics of each site regardless of the overall diagnosis of each patient, 12 gingivitis sites demonstrated a trend of more total Sclerostin in the GCF (148.18 ± 40.27 pg) (*p* = 0.09) when compared to 51 periodontitis sites (123.27 ± 19.08 pg) and 45 healthy sites (127.03 ± 46.86 pg) (Figure 1A). Alternatively, when considering the overall diagnosis of each patient group, the amount of total Sclerostin was similar in periodontitis, gingivitis and healthy sites of patients with periodontitis (*p* = 0.10) (Figure 1B), and it was also similar in gingivitis and healthy sites of patients diagnosed strictly with gingivitis (*p* = 0.30) (Figure 1C). The amounts of Sclerostin did not correlate with increasing PPD or CAL (*p* > 0.05) in any patient diagnostic category.

#### 3.3.2. WNT-5a Could Be Both: Osteoclastogenic and Pro-Osteoblastic

**Figure 2:**
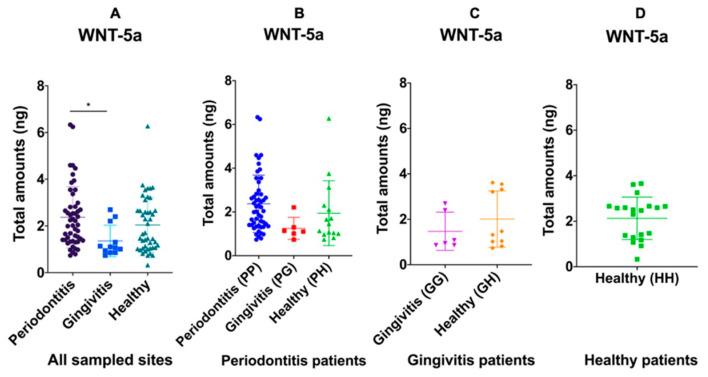
Distribution of the total amount of GCF WNT-5a in periodontitis, gingivitis and healthy sites regardless of the patient diagnosis (**A**); within the periodontitis patient group (**B**); gingivitis and healthy sites in the gingivitis patient group (**C**); and healthy sites in the healthy patient group (**D**). The solid lines show mean values. When all samples were considered, the total amount of GCF WNT-5a in gingivitis sites was significantly lower than in periodontitis and healthy sites (*p* = 0.03). Within the periodontitis (*p* = 0.11) and gingivitis (*p* = 0.32) patient groups, no significant differences were observed.

WNT-5a was also measured in the same total number of sites as Sclerostin. Irrespective of the overall diagnosis of each patient, total amounts of WNT-5a was similarly high in periodontitis sites (2.37 ± 1.31 ng) and healthy sites (2.04 ± 1.18 ng) while lower in gingivitis sites (1.36 ± 0.67 ng) (*p* = 0.03) (Figure 2A). Within each patient diagnostic category, total WNT-5a was similar among all types of sites in patients with periodontitis (*p* = 0.11) (Figure 2B) and in patients diagnosed with gingivitis (*p* = 0.32) (Figure 2C). The amounts of Wnt5a also did not correlate with increasing PPD or CAL (*p* > 0.05) in any patient diagnostic category.

With respect to smoking habits, SOST and WNT-5a GCF amounts demonstrated no significant differences between smokers and nonsmokers (*p* > 0.05). Similarly, when correlation models were utilized to assess the association between GCF amounts and patient’s age, no significant differences were found (*p* > 0.05).

## 4. Discussion

The total amounts of Sclerostin and WNT-5a in GCF samples of individuals with a healthy periodontium, gingivitis and generalized periodontitis were evaluated in this study. Considering the literature, this is the first investigation to address the total protein amounts of SOST and WNT-5a in gingivitis sites. GCF is a biological fluid that includes information about the physiological status of periodontal tissues and has been used for diagnostic purposes [18]. More than 90 different components, including inflammatory mediators, enzymes and tissue degradation products, have been evaluated in GCF [31]. It has widely been reported that the total protein amount in GCF samples per sampling time is more sensitive than concentrations due to GCF volume variability [32]. The variability in GCF volume between healthy and diseased periodontal tissues may affect the concentration of GCF components as sites with moderate and severe periodontitis may exhibit 10- to 30-fold higher GCF volume than healthy sites [33].

In a ligature-induced periodontitis study, an increased expression of SOST was associated with reduced bone formation, while increased bone formation was accompanied by a reduced expression of SOST-positive osteocytes [34]. Treatment with anti-Sclerostin antibody enhances alveolar bone mass, leads to the formation of cellular cementum and increases alveolar bone deposition [11,35]. Data from clinical studies have shown that SOST is upregulated in periodontal tissues, saliva and GCF of chronic periodontitis patients when compared to healthy sites [36,37,38,39]. In the present study, although there was a trend towards higher Sclerostin amounts in the gingivitis sites (*p* = 0.09), no significant differences were observed between periodontitis, gingivitis and healthy sites in periodontitis patients (*p* > 0.05). This may be attributed to the inclusion of an older population than the previous study and the analysis of samples taken from both anterior and posterior teeth rather than single-rooted teeth [37]. In agreement with our findings, Yakar et al. reported similar SOST levels in periodontitis and periodontal healthy groups [40].

Although periodontitis and healthy sites demonstrated similar Sclerostin amounts in GCF, gingivitis sites exhibited a trend towards higher amounts. Varying levels may reflect different stages of disease chronicity and activity. The higher levels in gingivitis sites could account for the preparation of this clinical form of disease to a more destructive condition leading to the initiation of bone loss. Protein levels of Sclerostin could be increased during the early period of tissue destruction, followed by a significant decrease when the initial stimulus has already disappeared in periodontitis sites. In addition, the lack of such a stimulus in healthy sites explains the similarly low levels between periodontitis and healthy periodontal sites.

WNT-5a has a dual role in the regulation of bone homeostasis by signaling different receptors that can either activate the canonical or the noncanonical pathway [16]. It suppresses bone formation by enhancing RANKL-induced osteoclastogenesis, and its upregulated secretion may also enhance the expression of inflammatory-related genes through a novel NF-κB-dependent regulation [12,14]. Studies have shown that WNT-5a is closely related to inflammatory bone resorption diseases such as rheumatoid arthritis, as well as inflammatory diseases such as sepsis and atherosclerosis [41,42]. *Porphyromonas gingivalis* LPS induces Wnt5a mRNA, revealing that WNT-5a plays an important role in periodontal inflammation [43]. In the gingiva of chronic periodontitis patients, Wnt5a is expressed significantly higher than in periodontally healthy patients [43]. The expression of Wnt5a is also significantly increased in gingival tissues of aggressive periodontitis when compared to tissues from chronic periodontitis [44]. Wnt5a is also significantly upregulated in gingival tissues of peri-implantitis patients in response to *Porphyromonas gingivalis* [45]. Inconsistencies between Wnt5a mRNA expression and GCF levels could be a result of a lag between the production of WNT-5a in the tissue and their release into the periodontal pocket.

In the present study, WNT-5a total amounts were significantly elevated at periodontitis sites when compared to gingivitis. In gingivitis sites, the WNT-5a levels are low, as the osteoclastogenic activity is not as pronounced as in periodontitis sites, where the osteoclastic activity is sustained. Within the periodontitis and gingivitis patient groups, WNT-5a amounts were similar between the different site groups. This can be attributed to the increased variability in WNT-5a production among individuals and the low number of individuals in the gingivitis patient group. The findings of our investigation also showed that the WNT-5a levels were similarly high between periodontitis and healthy sites. This can be explained by the inflammatory and osteoclastogenic activity of each site. As WNT-5a promotes osteoblast differentiation through the upregulation of LR5/6 {14,15}, WNT-5a sustains active osteoclastogenesis and bone destruction in an inflammatory environment (periodontitis sites), while in a noninflammatory environment of healthy sites, the pro-osteoblastic activity of WNT-5a prevails.

Our present findings show that Sclerostin is active and in high amounts at the initial stages of the disease when inflammation initiates osteoclastogenesis during gingivitis, while WNT-5a is osteoclastogenic and active during the chronic inflammatory process, which is associated with active bone destruction in periodontitis. Therefore, Sclerostin may be a promising prognostic marker of disease, whereas WNT-5a can possibly be utilized as a diagnostic marker. This study was used to generate a hypothesis and ultimately provide evidence to initiate further studies, including longitudinal investigations. Differences between patient groups in regards to age and smoking habits are also considered limitations of this study. WNT signaling is associated with aging, and the expression of WNT genes was shown to be decreased in adult and old mice when compared to a young group of mice [46]. Decreased expression of WNT-5a was also observed in mature osteoblasts of aged mice demonstrating the importance of an individual’s age [45]. Smoking may also affect the production of GCF and GCF levels of proinflammatory cytokines [47,48]. In the present study, smokers were only found in the periodontitis patient group. However, when Sclerostin and WNT-5a GCF amounts were compared between smokers and nonsmokers, no significant differences were observed (*p* > 0.05). Finally, future studies should include equal race sample sizes to seek a representation of minority groups. In the present investigation, the low number of African Americans, Asians and Hispanics can be attributed to the relatively low attendance of these minority groups at the School of Dentistry.

## 5. Conclusions

In conclusion, Sclerostin and WNT-5a were detectable in the GCF of periodontitis, gingivitis and healthy patients. WNT-5a levels in GCF depend on the level of disease of periodontitis sites. The WNT-5a total amounts in the GCF of gingivitis sites were significantly lower than in periodontitis sites. Sclerostin trended higher in gingivitis but was similar in periodontitis and healthy sites. Within each patient disease group, the total amounts of Sclerostin and WNT-5a were similar among sites with different disease severity. Studies investigating the GCF levels of Sclerostin and WNT-5a after periodontal therapy may further elucidate the role of these molecules in the pathogenesis of periodontal disease and evaluate their potential diagnostic and prognostic values.

## Figures and Tables

**Table 1:** Patient characteristics and full-mouth clinical periodontal parameters of study groups.

Demographic, Smoking and Clinical Characteristics	Periodontitis Group (*N* = 20–45.5%)	Gingivitis Group (*N* = 10–22.7%)	Healthy Group (*N* = 14–31.8%)	Total Population (*N* = 44–100%)	*p*-Value *
**Age**					<0.001 ^†^
Mean (SD)	58.7 (13.8)	31.6 (12.7)	27.1 (8.4)	42.5 (19.1)	
**Gender**					0.21 ^‡^
Males (%)	10 (50)	2 (20)	4 (28.6)	16 (36.4)	
Females (%)	10 (50)	8 (80)	10 (71.4)	28 (63.6)	
**Race**					0.13 ^‡^
Caucasians (%)	14 (70)	10 (100)	11 (78.6)	35 (79.5)	
African Americans (%)	5 (25)	0 (0)	1 (7.1)	6 (13.6)	
Asians (%)	0 (0)	0 (0)	2 (14.3)	2 (4.5)	
Hispanics (%)	1 (5)	0 (0)	0 (0)	1 (2.3)	
**Smoking status**					<0.001 ^‡^
Never smokers (%)	6 (30)	10 (100)	14 (100)	30 (68.2)	
Ex-smokers (%)	6 (30)	0 (0)	0 (0)	6 (13.6)	
Current smokers (%)	8 (40)	0 (0)	0 (0)	8 (18.2)	
**Amount of smoking**					1.00 ^‡^
<10 cig/day (%)	0 (0)	0 (0)	0 (0)	0 (0)	
10–20 cig/day (%)	2 (25)	0 (0)	0 (0)	2 (25)	
>20 cig/day (%)	6 (75)	0 (0)	0 (0)	6 (75)	
**Missing teeth** (mean ± SD)	4.90 ± 3.71	0.80 ± 1.14	0.79 ± 1.58	2.66 ± 3.38	<0.001 ^†^
**Full mouth** (mean ± SD)					
PPD (mm)	3.82 ± 1.20	2.15 ± 0.31	1.70 ± 0.22	2.77 ± 1.28	<0.001 ^†^
CAL (mm)	3.95 ± 1.69	0.00 ± 0.00	0.00 ± 0.00	1.80 ± 2.28	<0.001 ^†^
BOP (%)	45.65 ± 31.02	25.50 ± 18.78	4.21 ± 2.12	27.89 ± 28.82	<0.001 ^†^
PI (%)	30.39 ± 34.33	4.90 ± 6.03	1.21 ± 2.72	15.31 ± 26.95	0.002 ^†^
Sites with PPD = 1–3 mm (%)	55.56 ± 21.45	95.20 ± 6.67	99.31 ± 0.73	78.49 ± 25.75	<0.001 ^†^
Sites with PPD = 4–6 mm (%)	33.08 ± 14.55	4.80 ± 6.67	0.00 ± 0.00	16.13 ± 18.74	<0.001 ^†^
Sites with PPD ≥ 7 mm (%)	10.98 ± 15.26	0.00 ± 0.00	0.00 ± 0.00	4.99 ± 11.55	0.005 ^†^
Sites with CAL = 0–2 mm (%)	33.46 ± 21.40	100.00 ± 0.00	100.00 ± 0.00	69.76 ± 36.41	<0.001 ^†^
Sites with CAL = 3–4 mm (%)	32.46 ± 10.94	0.00 ± 0.00	0.00 ± 0.00	14.76 ± 17.89	<0.001 ^†^
Sites with CAL ≥ 5 mm (%)	34.08 ± 24.98	0.00 ± 0.00	0.00 ± 0.00	15.49 ± 23.88	<0.001 ^†^

* Statistical significance between study groups with a value of *p* < 0.05. ^†^ ANOVA was used to compare the mean values between the study groups. ‡ Chi-square tests were used to compare categorical variables including gender, race, smoking status and amount of smoking between the study groups. Abbreviations: SD: Standard deviation; PPD: probing pocket depth; CAL: clinical attachment level; BOP: bleeding on probing; PI: plaque index.

**Table 2:** Periodontal characteristics of the periodontitis, gingivitis and healthy sampled sites regardless of the patient diagnosis.

Clinical Characteristics	Periodontitis Sites (*N* = 51)	Gingivitis Sites (*N* = 12)	Healthy Sites (*N* = 45)	*p*-Value *
PPD (mean ± SD)	6.41 ± 2.05	2.67 ± 0.49a	2.31 ± 0.73a	<0.001
CAL (mean ± SD)	6.75 ± 2.51	0.92 ± 1.08a	0.62 ± 1.50a	<0.001
BOP (%)	43 (84.3)	12 (100.0)	0 (0.0)	<0.001
Sites with PPD = 1–3 mm (%)	0 (0.0) ^†^	12 (100.0)	45 (100.0)	<0.001
Sites with PPD = 4–6 mm (%)	31 (60.8)	0 (0.0)	0 (0.0)	
Sites with PPD ≥ 7 mm (%)	20 (39.2)	0 (0.0)	0 (0.0)	
Sites with CAL = 0–2 mm (%)	0 (0.0) ^†^	12 (100.0)	45 (100)	<0.001
Sites with CAL = 3–4 mm (%)	8 (15.7)	0 (0.0)	0 (0.0)	
Sites with CAL ≥ 5 mm (%)	43 (84.3)	0 (0.0)	0 (0.0)	

* Statistical significance between study groups with a value of *p* < 0.05. ^†^ By definition, periodontitis sites did not include sites with PPD = 1–3 mm and CAL = 0–2 mm. Abbreviations: PPD: probing pocket depth; CAL: clinical attachment level; BOP: bleeding on probing.

**Table 3:** Periodontal clinical characteristics of the sampled sites in periodontitis, gingivitis and healthy patient groups.

	Periodontitis Patient Group ^†^			Gingivitis Patient Group ^†^		Healthy Patient Group ^†^
Clinical Characteristics	Periodontitis Sites PP (*N* = 51)	Gingivitis Sites PG (*N* = 6)	Healthy Sites PH (*N* = 15)	Gingivitis Sites GG (*N* = 6)	Healthy Sites GH (*N* = 10)	Healthy Sites HH (*N* = 20)
PPD (mean ± SD)	6.41 ± 2.05	2.5 ± 0.55 ^a^	2.47 ± 0.74 ^a^	2.83 ± 0.41	2.5 ± 0.85	2.10 ± 0.64 ^a^
CAL (mean ± SD)	6.75 ± 2.51	1.83 ± 0.75 ^a^	1.87 ± 2.13 ^a^	0.00 ± 0.00	0.00 ± 0.00	0.00 ± 0.00 ^a^
BOP (%)	43 (84.3)	6 (100.0)	0 (0.0)	6 (100.0)	0 (0.0)	0 (0.0)
Sites with PPD = 1–3 mm (%)	0 (0)	6 (100.0)	15 (100.0)	6 (100.0)	10 (100.0)	20 (100.0)
Sites with PPD = 4–6 mm (%)	31 (60.8)	0 (0.0)	0 (0.0)	0 (0.0)	0 (0.0)	0 (0.0)
Sites with PPD ≥ 7 mm (%)	20 (39.2)	0 (0.0)	0 (0.0)	0 (0.0)	0 (0.0)	0 (0.0)
Sites with CAL = 0–2 mm (%)	0 (0.0)	6 (100.0)	15 (100.0)	6 (100.0)	10 (100.0)	20 (100.0)
Sites with CAL = 3–4 mm (%)	8 (15.7)	0 (0.0)	0 (0.0)	0 (0.0)	0 (0.0)	0 (0.0)
Sites with CAL ≥ 5 mm (%)	43 (84.3)	0 (0.0)	0 (0.0)	0 (0.0)	0 (0.0)	0 (0.0)

^†^ Comparison of clinical characteristics within each patient group. (a) Periodontitis patient group: periodontitis (PP) vs. gingivitis (PG) vs. healthy (PH) sites; (b) gingivitis patient group: gingivitis (GG) vs. healthy (GH) sites. ^a^ Significant difference (*p* < 0.001) of gingivitis and healthy sites compared with periodontitis sites. Abbreviations: PPD: probing pocket depth; CAL: clinical attachment level; BOP: bleeding on probing.

## Data Availability

The data presented in this study are available on request from the corresponding author.
